# Supplementary material for: Activating reversible carbonate reactions in Nasicon solid electrolyte-based Na-air battery via in-situ formed catholyte
Source: Nat Commun. 2024 Apr 5;15:2952. doi: 10.1038/s41467-024-47415-0 (PMC10997774; doi:10.1038/s41467-024-47415-0)
Supplement: Supplementary file 1 — Supplementary Information [file 41467_2024_47415_MOESM1_ESM.pdf]

Supplementary Information

Activating reversible carbonate reactions in Nasicon solid electrolyte-based Na-air battery via in-situ formed catholyte

Heetaek Park[1], Minseok Kang[1], Donghun Lee[1], Jaehyun Park[2], Seok Ju Kang[2], Byoungwoo Kang[1,*]

[1]Department of Materials Science and Engineering, Pohang University of Science and Technology (POSTECH), 77 Cheongamro, Namgu, Pohang, Gyeongbuk, 37673, South Korea

[2]Department of Energy Engineering School of Energy and Chemical Engineering Ulsan National Institute of Science and Technology (UNIST) 50 UNIST-gil, Ulsan 44919, South Korea

*Corresponding author. Tel: +82 54 279 2154

E-mail address:bwkang@postech.ac.kr (B. Kang)

1. List of possible electrochemical/chemical reactions

Several electrochemical/chemical reactions are possible in Na-air batteries (Tables S1, S2).

Table S1. List of possible chemical reactions and their Gibbs formation energy (Ref: Principles of Modern Chemistry, Science Plus, 7th edition, 2014)

Reaction	ΔG_f (kJ mol^{-1})
$2NaO_2$ (s) + C (s) → Na_2CO_3 (s) + $0.25O_2$ (g)	-606.97
2NaOH (s) + C (s) + O_2 (g) → Na_2CO_3 (s) + H_2O (l)	-522.61
NaOH (s) + CO_2 (g) → $NaHCO_3$ (s)	-77.147
NaOH (aq) + CO_2 (g) → $NaHCO_3$ (aq)	-35.167
2NaOH (aq) + CO_2 (g) → Na_2CO_3 (aq) + H_2O (l)	-213.336
2NaOH (s) + CO_2 (g) → Na_2CO_3 (s) + H_2O (l)	-128.222
2NaOH (s) + CO_2 (g) → Na_2CO_3 (aq) + H_2O (l)	-292.632
Na_2CO_3 (s) + CO_2 (g) + H_2O (g) → $2NaHCO_3$ (s)	-34.629
Na_2CO_3 (s) + CO_2 (g) + H_2O (l) → $2NaHCO_3$ (s)	-26.072
Na_2CO_3 (s) + H_2O (g) → $Na_2CO_3 \cdot H_2O$ (s)	-12.335
NaOH + H_2O (g) → $NaOH \cdot H_2O$	-21.428

Table S2. Lists of possible electrochemical reactions, their Gibbs formation energy (Ref: Principles of Modern Chemistry, Science Plus, 7th edition, 2014), and their redox potentials

Reaction	ΔG_f (kJ mol^{-1})	E (V)
$2Na + CO_2\ (g) + 0.5O_2\ (g) \rightarrow Na_2CO_3\ (s)$	-650.081	3.37
$2Na + CO_2\ (g) + 0.5O_2\ (g) \rightarrow 2Na^+\ (aq) + CO_3^{2-}\ (aq)$	-657.261	3.41
$2Na + CO_2\ (aq) + 0.5O_2\ (g) \rightarrow Na_2CO_3\ (s)$	-658.46	3.41
$2Na + CO_2\ (aq) + 0.5O_2\ (g) \rightarrow 2Na^+\ (aq) + CO_3^{2-}\ (aq)$	-665.64	3.45
$2Na + CO_2\ (g) + 0.5O_2\ (g) + H_2O\ (g) \rightarrow Na_2CO_3{\cdot}H_2O\ (s)$	-662.418	3.43
$Na + 0.5H_2O\ (g) + 0.25O_2\ (g) \rightarrow NaOH\ (s)$	-265.208	2.75
$Na + 1.5H_2O\ (g) + 0.25O_2\ (g) \rightarrow NaOH{\cdot}H_2O\ (s)$	-286.636	2.97
$Na + 1.5H_2O\ (l) + 0.25O_2\ (g) \rightarrow NaOH{\cdot}H_2O\ (s)$	-273.625	2.84
$Na + 0.5H_2O\ (l) + 0.25O_2\ (g) \rightarrow Na^+\ (aq) + OH^-\ (aq)$	-300.5885	3.12
$Na + 0.5H_2\ (g) + CO_2\ (g) + 0.5O_2\ (g) \rightarrow NaHCO_3\ (s)$	-456.641	4.73
$Na + 0.5H_2\ (g) + CO_2\ (g) + 0.5O_2\ (g) \rightarrow Na^+\ (aq) + HCO_3^-\ (aq)$	-454.316	4.71
$Na + H_2O\ (l) + CO_2\ (g) \rightarrow Na^+\ (aq) + HCO_3^-\ (aq) + 0.25H_2\ (g)$	-217.187	2.25

2. Material properties of the dense Nasicon ($Na_3Zr_2Si_2PO_{12}$) solid electrolyte (SE)

The dense Nasicon SE was obtained by sintering for 10 h at 1100 °C. The pellet density was 3.0 g cm^{-3} (~ 9 % porosity) and the total ionic conductivity was 2.1×10^{-4} S cm^{-1} at RT (Fig. S1).

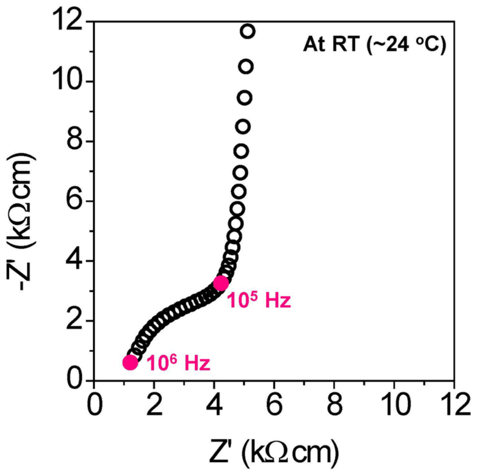

Figure S1. Nyquist plot of the dense Nasicon SE measured at RT.

3. Ni metal as an electrochemically stable current collector

To understand the reaction of water with metal current collectors, a reaction between DI water (1 ml) and the air-electrode was investigated by performing linear sweep voltammetry tests (Fig. S2a). The scanning rate was 1 mV s^{-1}. The current increased abruptly at ~ 3.25 V in the cell with the Ag current collector, but was relatively low up to 3.9 V in cells with Ni or Au current collectors; 3.9 V is the water-decomposition potential. An XRD spectrum (Fig. S2b) was collected from the air-electrode after the linear sweep test. Although high current was obtained by charging the cell to 5 V, the components in the air-electrode did not show any

decomposition. Also, it should be noted that much lower amount of water was included in the air-electrode during electrochemical cycle in ambient air. Therefore, we concluded that the electrochemical reactions of the SE-based Na-air battery were obtained by redox reactions of Na compounds, rather than side reactions between the absorbed water and the air-electrodes or current collector metals.

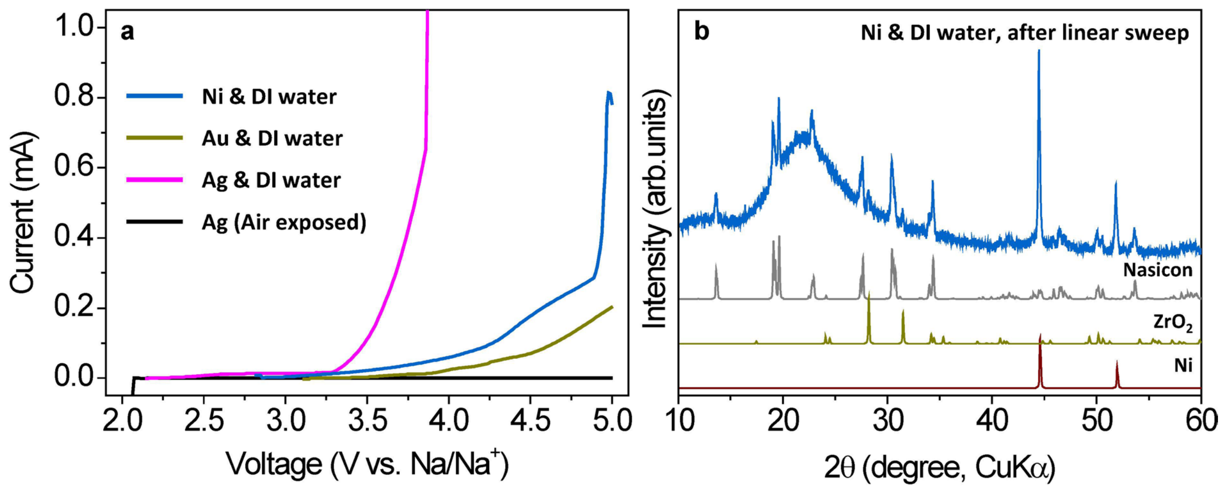

Figure S2. Electrochemical stability influenced by various metal current collectors. a, Linear sweep voltammetry tests of the SE-based Na–air cells with different metal current collectors (Ag, Au, and Ni). **b,** XRD data of the air-electrode with Ni current collector after linear sweep voltammetry test.

4. Effects of Au layer between Na metal and dense Nasicon SE

A gold (Au) film was deposited on the dense Nasicon ($Na_3Zr_2Si_2PO_{12}$) electrolyte toward the Na metal anode. To understand the effects of the Au on interfacial resistance between Na metal and Nasicon SE, symmetric cells were prepared and their electrochemical properties during charge/discharge were measured using electrochemical impedance spectroscopy (EIS)

(Fig. S3). The Au thin film substantially reduced the interfacial resistance. Considering that the Au layer reduces interfacial resistance between Li metal and $Li_7La_3Zr_2O_{12}$ (LLZO)[1], possibly because of formation of Li-Au alloy that enables to development of homogeneous contact between Li metal and LLZO solid electrolyte, and Na-Au alloy is thermodynamically stable[2,3], we presume that the Au layer can have a similar function here as was suggested in the previous Li studies. Therefore, Na-Au alloy forms, and can induce homogeneous contact between dense Nasicon SE and Na metal resulting in substantially reduced interfacial resistance.

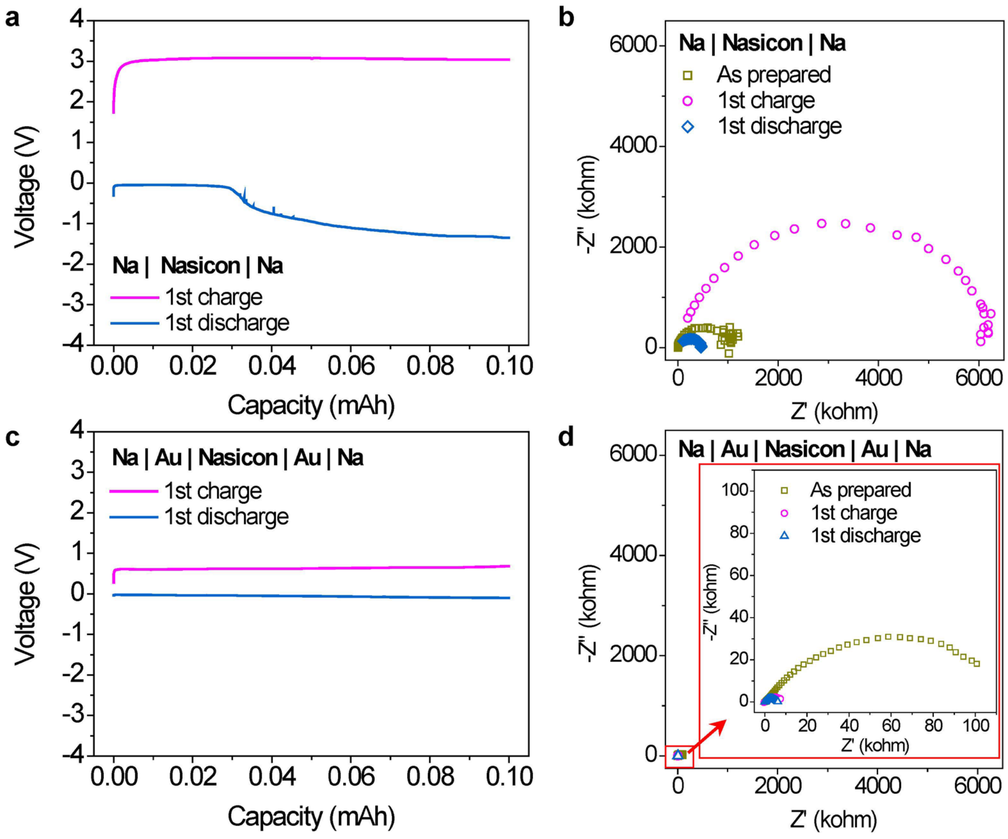

Figure S3. Interfacial resistance between Na metal and dense Nasicon solid electrolyte. **a,** Voltage profiles, **b,** Nyquist plots of Na –Nasicon symmetrical cells without Au buffer layer,

c, Voltage profiles, and **d**, Nyquist plots of Na –Nasicon symmetrical cell with Au buffer layer. The symmetric cells were tested at a current density of 0.02 mA cm^{-2} within 0.1 mAh cm^{-2}.

5. Cell structure of the Nasicon SE based Na-air batteries

The SE-based Na-air cell (Fig. S4) is sandwiched between a stainless-steel current collector for the anode side and stainless-steel plate for the air-electrode side.

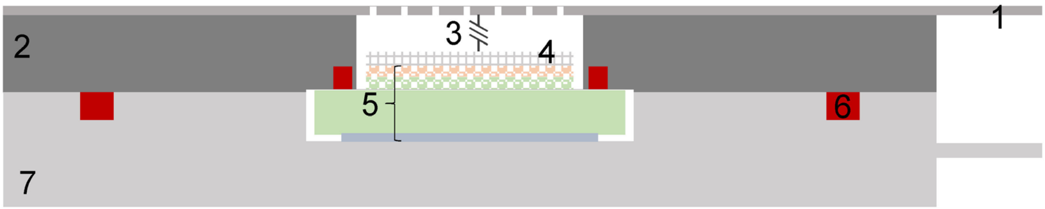

1. Stainless plate (cathode side)
2. Polycarbonate cover
3. Spring
4. Pt mesh
5. Air cell (Ni current collector | duplex | Au | Na metal)
6. Sealant
7. Stainless current collector (anode side)

Figure S4. Schematic design of the cell hardware for the SE-based Na-air battery

6. Protection of the Na metal anode

After assembling the SE-based Na-air cells, the O_2 gas were blown for 3 h toward the air-electrode side of the SE-based Na-air cell ('4' in Fig. S4), and then the cells were tested (Fig. S5a). After blowing O_2 gas, the cycle life of the cell was extended from 37 cycles (no gas flow) to 53 cycles (99.995 % O_2 flow) (Fig. S5b). We speculate that the cell death might be caused by the contamination of Na metal anode induced by the permeation of air, especially water through an imperfect seal ('6' in Fig. S4), or through pores in the dense Nasicon SE ('5' in Fig.

S4), or by both mechanisms. Therefore, we tried to suppress the contamination of the Na metal with a protection layer by using chemical reactions between pure O_2 gas and Na metal as has been already reported in Li-air batteries: a passivation layer (Li_2CO_3) was formed electrochemically on the Li anode by cycling Li-CO_2 batteries.[4] The result suggests that the blow of O_2 gas before electrochemical test suppresses the fast degradation of Na metal anode, and enables stable extraction/deposition of Na metal during electrochemical tests. It might be possible that a new layer (probably Na oxides) was formed by a reaction between oxygen and Na. However, further clarification or study can be required for this observation.

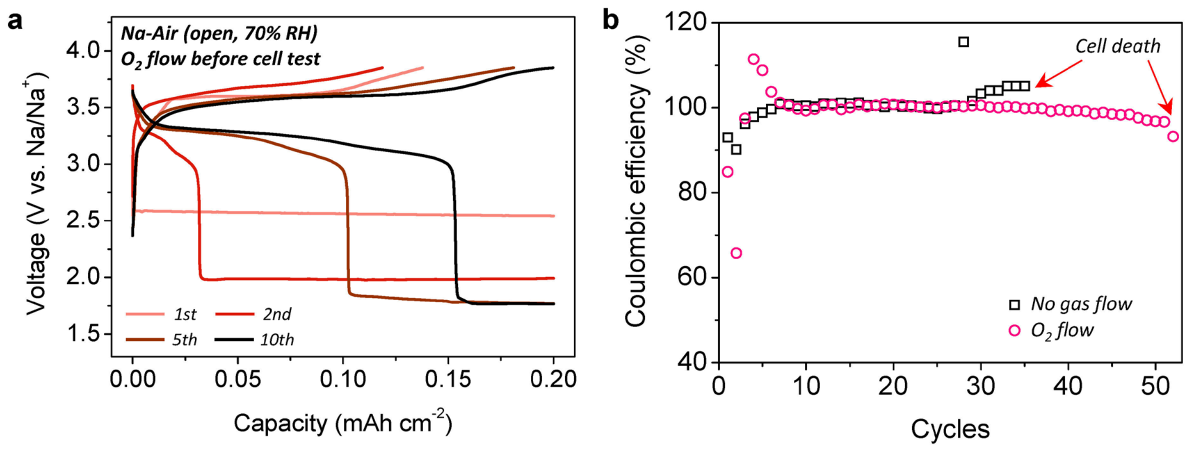

Figure S5. Effects of H_2O-free gas pre-treatment on the cyclability of the SE-based Na-air cells. a, Voltage profiles after blowing O_2 gas before cell tests. **b,** Change in coulombic efficiency over cycles at a current density of 0.1 mA cm^{-2}. All cell tests were conducted in air with 70% RH at 25 °C. The cells were discharged to 0.2 mAh cm^{-2} and charged up to 3.85 V. The initial cycle employed a current density of 0.02 mA cm^{-2}, followed by 0.1 mA cm^{-2} for the remaining cycles.

7. Increase in the electrochemical activity by the presence of the moisture in air

To understand the effect of the H_2O in the air on the electrochemical reactions, the SE-based Na-air cells were operated in various atmospheres (Fig. S6a) with the current density of 0.02 mA cm^{-2}. When high concentrations of reactive gases such as pure O_2, pure CO_2, pure N_2, 21% O_2/79% N_2, or 33% O_2/67% CO_2 were supplied (i.e., water vapor was extremely low or absent), the electrochemical activities of the cells were negligible. The cells showed high electrochemical activity only when they are tested under humid air that allows the electrochemical formation of NaOH (Fig. 2 in the manuscript). These results indicate that the NaOH reaction (equation 2 in the manuscript) is more active than the other electrochemical reactions that can yield sodium oxides and carbonates (Table S2). Furthermore, considering that carbonate-related reactions at ~ 3.4 V observed in ambient air with 70% RH (Fig. 2 in the manuscript) are activated but they are negligible in electrochemical dry reactant gas of 67% CO_2 and 33% O_2, the existence of H_2O that can increase the CO_2 concentration near reaction sites seems very critical to activate the electrochemical reactions of the carbonates.

The SE-based Na-air battery was also investigated using a cyclic voltammetry measurement in ambient air with 70% RH at 25 °C (Fig. S6b). The CV curve showed reduction peaks at < 2.5 V and < 3.4 V, respectively and oxidation peaks at > 3.5 V as the cycle increases. It is noteworthy that this behavior is consistent with the voltage profile obtained in the constant current condition (Fig. 2 and 4 in the manuscript) and the electrochemical activity is gradually increased by repeating cycles. The result can demonstrate that the $Na_2CO_3 \cdot xH_2O$ reaction is electrochemically activated and the amount of the in-situ formed catholyte is increased by repeating cycles.

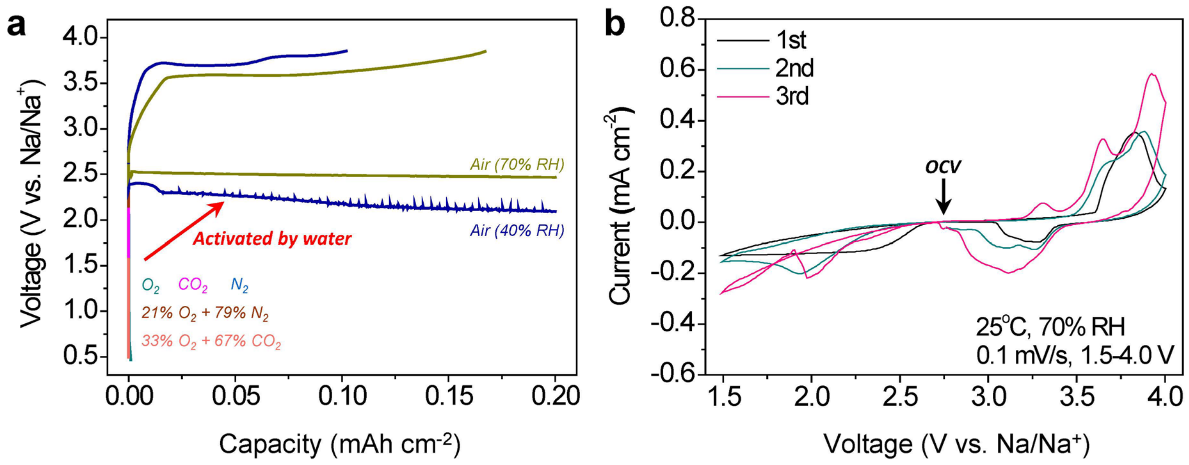

Figure S6. Effects of humid air on the electrochemical activity of the SE-based Na-air cells. a, Electrochemical activities of the SE-based cells at 1st cycle operated under various atmospheres (pure O_2, pure CO_2, pure N_2, 21% O_2 + 79% N_2, 67% CO_2 + 33% O_2, air with 40% or 70% RH) at 25 °C. The cells were discharged to 0.2 mAh cm^{-2}, then charged to 3.85 V at a current density of 0.02 mA cm^{-2}. **b,** Cyclic voltammogram of the SE-based Na-air battery at a scan rate of 0.1 mV s^{-1} under ambient air with 70% RH at 25 °C within the voltage range of 1.5 - 4.0 V.

8. Electrochemical properties of the Nasicon SE-based Na-air batteries

8-1. Voltage profiles of the air-cathodes for ex-situ XRD/SEM measurements

Fig. S7 shows the discharge-charge voltage profiles of the cells of Fig. 2b-c and Fig. 3a-c in the manuscript. The coulombic efficiency of the charged cell (Fig. S7b) was 82.5%; this discrepancy in capacity between charge/discharge corresponds to 0.15 mg of NaOH and 0.16 mg of $Na_2CO_3{\cdot}H_2O$, respectively. The quantity of remaining products was extremely small compared to air electrodes (3 ~ 4 mg), so XRD peaks of NaOH and $Na_2CO_3{\cdot}H_2O$ might not be detected in the charged air-electrode.

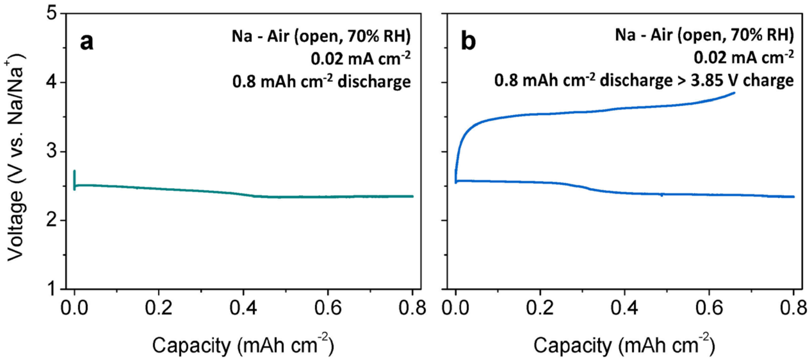

Figure S7. Voltage profiles of the SE-based Na-air cells for ex-situ XRD/SEM measurements. a-b, Voltage profiles of the (a) discharged and (b) charged cells in 1st cycle for ex-situ XRD/SEM measurements (Fig. 2b-c and Fig. 3a-c in manuscript). All tests were conducted in air with 70% RH at 25 °C. Current density was 0.02 mA cm^{-2}, and cutoff conditions of discharge and charge were 0.8 mAh cm^{-2} and 3.85 V, respectively.

8-2. Ex-situ/in-situ analysis for electrochemical mechanism of the SE-based Na-air cell

The top view of the air-electrodes was observed after by ex-situ Raman spectra (Fig. S8). We note that the cell was charged and discharged with the same condition as the cell in Fig. S7 but was additional one different from the cell of Fig. S7. As shown in ex-situ XRD (Fig. 2b in the manuscript), the Raman band at ~1080 cm^{-1} also shows that the $Na_2CO_3 \cdot xH_2O$ is formed and decomposed during cycling of the SE-based Na-air cell.[5]

We performed in-situ DEMS analysis of the 1st charge reaction of Na-air battery (Fig. S9). Firstly, the Na-air cell was discharged to 0.2 mAh cm^{-2} in air with 70% RH, and then, a closed space with a quartz tube and gas lines was added to the cathode side of the cell hardware as shown in Fig. S9a. After that, the closed space of the cathode part was filled with Ar gas. Then, the Na-air cell was charged to 3.85 V and the evolution rate of CO_2 gas was measured at the

same time (Fig. S9b). The in-situ DEMS clearly shows that the CO_2 gas was generated during charge. Considering the applied current (0.02 mA cm^{-2}) and the Na_2CO_3 reaction (equation 5 in the manuscript) and the Faraday's law of electrolysis, the theoretical evolution rate of CO_2 gas during charge is 3.1 nmol min^{-1}, but the observed rate was ~1 nmol min^{-1}. This discrepancy would be originated from the dissolution of CO_2 in the absorbed water of the air-electrode. Therefore, after 1st cycle, the air-electrode of Na-air battery has CO_2-rich condition, leading to the Na_2CO_3 formation reaction during the following discharge step. It should be noted that a cell potential for in-situ DEMS measurement is lower than that of normal cell and it might be caused by contamination of Na anode due to non-perfect sealing.

During the discharge process, a mixed gas (O_2, CO_2, H_2O), similar to ambient air, should be flowed into the cell for the electrochemical reaction. However, the DEMS instrument what we used can only analyze the variance in the entire amount of mixed gas, not that of each gas. Therefore, ex-situ XRD and Raman analysis were performed to observe the proposed electrochemical reaction mechanism (Fig. S10). When the SE-based Na-air cell was discharged to 2.8 V, the formation of the discharge products ($Na_2CO_3 \cdot xH_2O$) was observed by Raman, not XRD. It might be originated by a small quantity of the discharge products. The CO_3^{2-} band of Na_2CO_3 (~ 1080 cm^{-1}) and OH bands of H_2O (3200 - 3500 cm^{-1}) was detected in the cell discharged to 2.8 V.[5, 6] When the discharge process is further proceeded at low potential, the intensity of XRD peaks corresponding to NaOH increased compared to those of $Na_2CO_3 \cdot xH_2O$ and Nasicon. The similar result was also observed in the ex-situ Raman analysis of the hybrid Na-air cell (Fig. S16). Combining these results, we can conclude that the $Na_2CO_3 \cdot xH_2O$ and NaOH is electrochemically formed at high (~3.4 V) and low (~2.0 V) potential, respectively.

Lastly, we performed in-situ Raman analysis to confirm direct formation of $Na_2CO_3 \cdot xH_2O$ during discharge (Fig. S11). We prepared new cell hardware for in-situ Raman measurement

(Fig. S11a), and the top of the Ni current collector was observed. We note that the high cell polarization was observed due to the uncontrolled and low humidity in the environment (Fig. S11b). The cell was activated for 5 cycles in air with 70% RH before the in-situ Raman measurement. It is noteworthy that the peak at ~1080 cm^{-1} corresponding to Na_2CO_3 grows during discharge (1~4 in Fig. S11c) is exhibited and then disappears by charging the cell (5~8 in Fig. S11d). The result demonstrates that $Na_2CO_3 \cdot xH_2O$ is electrochemically formed during discharge.

Differing from the ex-situ Raman experiment results, we did not observe any changes in the NaOH peak during the in-situ Raman experiment. This might be attributed to the fact that, unlike the ex-situ Raman measurement, which observed the entire electrode, the in-situ Raman experiment focused solely on the upper part of the electrode.

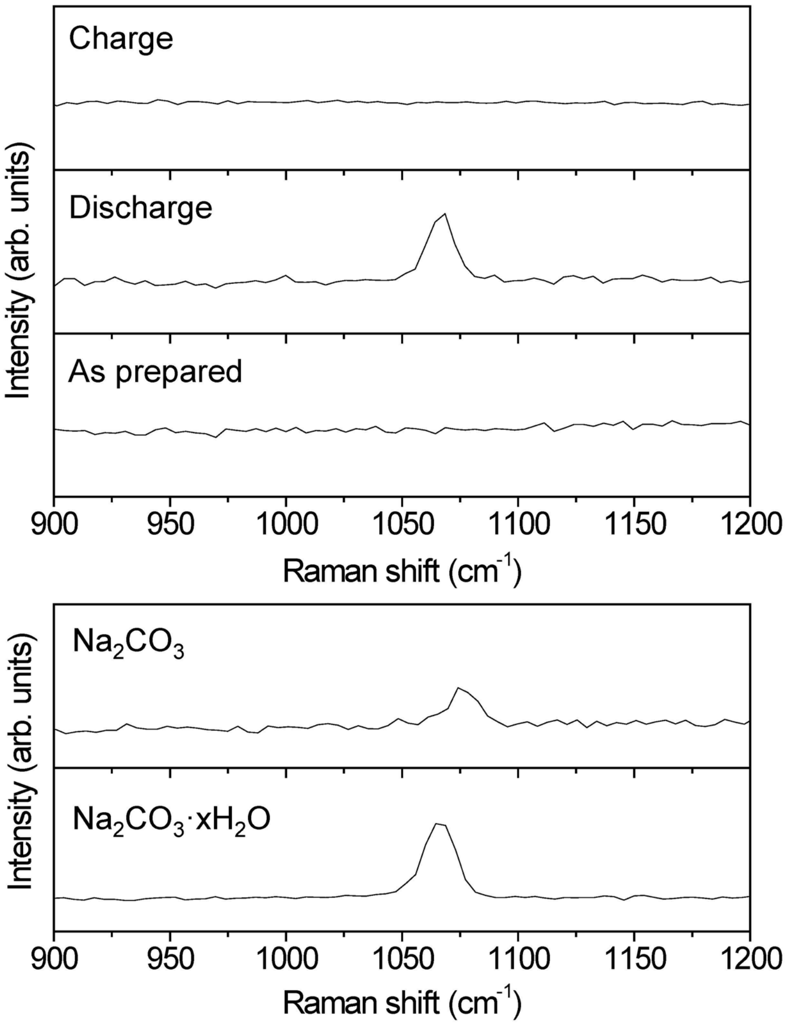

Figure S8. Ex-situ Raman spectra of air-electrodes in a pristine state, after 1st discharge, and after 1st charge.

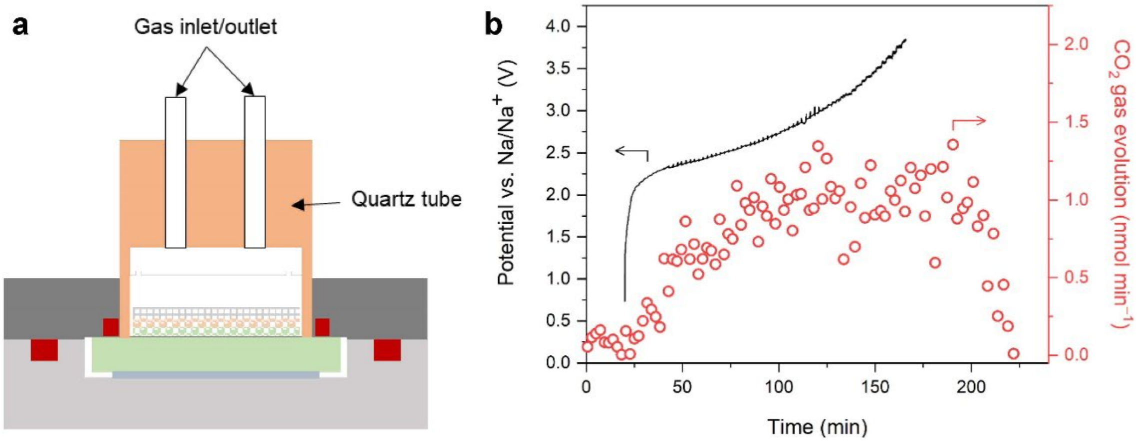

Figure S9. Quantification of evolved CO_2 gas during charge. a, Schematic of the DEMS electrochemical cell. **b,** In-situ DEMS data of the cell during 1st charge where the cell was charged to 3.85 V at a current density of 0.02 mA cm^{-2}.

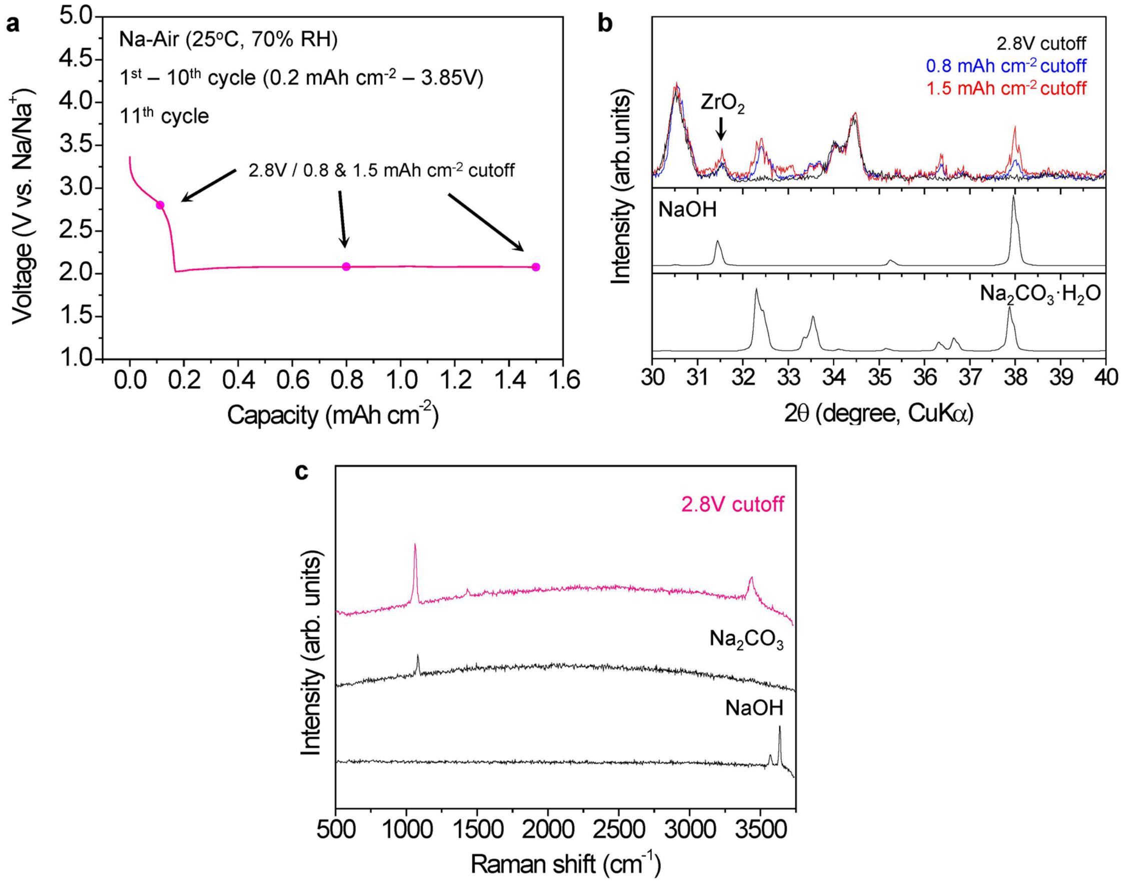

Figure S10. Investigation of redox mechanism via ex-situ analysis of discharge products. a, Voltage profiles of the SE-based Na-air cell during 11th cycle, discharged to 0.8

and 1.5 mAh cm^{-2} at a current density of 0.1 mA cm^{-2}. **b,** Ex-situ XRD patterns of the air-electrode under different discharge cutoff conditions: 2.8 V, 0.8 and 1.5 mAh cm^{-2}. **c,** Ex-situ Raman spectra of air-electrodes after discharging to 2.8 V. We note that the cell was pre-activated for 10 cycles by discharging to 0.2 mAh cm^{-2} and charging up to 3.85 V at a current density of 0.1 mAh cm^{-2}.

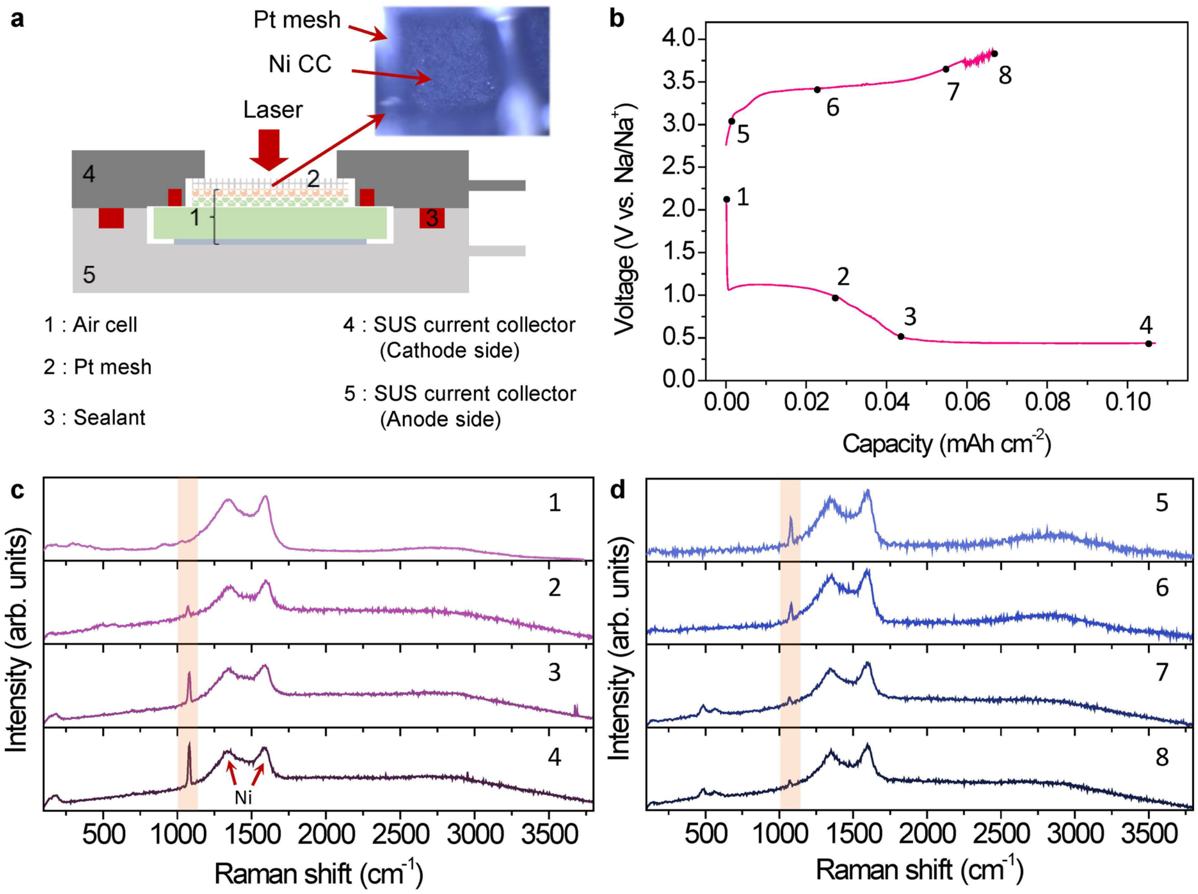

Figure S11, In-situ Raman analysis of the SE-based Na-air cells. a, Design of in-situ Raman cells (inset: optical image of upper-side of the air-electrode). **b,** Voltage profile of the SE-based Na-air cell during in-situ Raman test (current density: 0.1 mA cm^{-2}). **c-d,** In-situ Raman spectra observed (c) during discharge and (d) during charge. The cell was pre-activated for 5 cycles in air with 70% RH before the in-situ Raman measurement.

8-3. Change in microstructure of the air-electrodes caused by electrochemical test (Top view)

The top view of the air-electrodes was observed after cycling the SE-based Na-air cell (Fig. S12). Similarly to the cross-section view (Fig. 3a-c in the manuscript), the whole air-electrode was covered by film-like products after the discharge, and the products were removed after the charge.

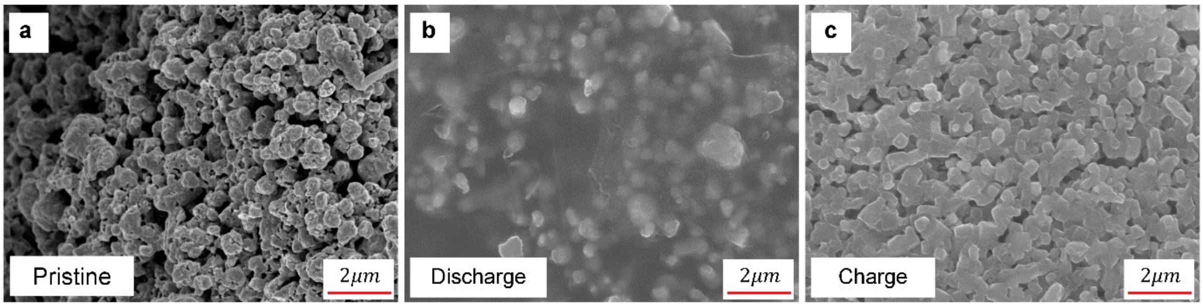

Figure S12. Microstructure changes in air-electrodes during cycling the SE-based Na-air cells. a-c, SEM images (top view) of air-electrodes in (a) pristine state, (b) after 1st discharge (0.8 mAh cm^{-2} discharge), and (c) after 1st charge (0.8 mAh cm^{-2} cut-off for the discharge and 3.85 V cut-off for the charge). Cell tests were conducted in air with 70% RH at 25 °C. The cycling conditions were identical to those in Fig. S7.

8-4. Electrochemical reactions of the Na-air cell after 10th cycle

To comfirm reversibility of the electrochemical reactions, the ex-situ analyses were performed after 10th cycle (Fig. S13). The cells were cycled for 10 times to fully activate the electrochemical reaction of the $Na_2CO_3 \cdot xH_2O$. Then, the cells were discharged to 0.8 mAh cm^{-2} and charged to 3.85 V during 11th cycle. The current densities for the test were 0.05 and 0.2 mA cm^{-1} at 1st and subsequent cycles, respectively (Fig. S13a). The ex-situ XRD/Raman/SEM data of the air-electrode at 11th cycle show that the discharge products were NaOH and

$Na_2CO_3{\cdot}xH_2O$ and are electrochemically formed and decomposed by cycling the cells as it did during 1st cycle (Fig. 2 in manuscript). The Raman band at 1080 cm^{-1} proves that the $Na_2CO_3{\cdot}xH_2O$ is electrochemically formed during the discharge of 11th cycle.[5] In addition, the Raman band at 3633 cm^{-1} corresponding unhyrdous NaOH (orange line in Fig. S13c)[6] in the discharged cell was almost diminished implying that NaOH was transformed to $Na_2CO_3{\cdot}xH_2O$ due to chemical reaction with air. The results indicate that the electrochemical reactions of $Na_2CO_3{\cdot}xH_2O$ and NaOH continuously occur during every cycle.

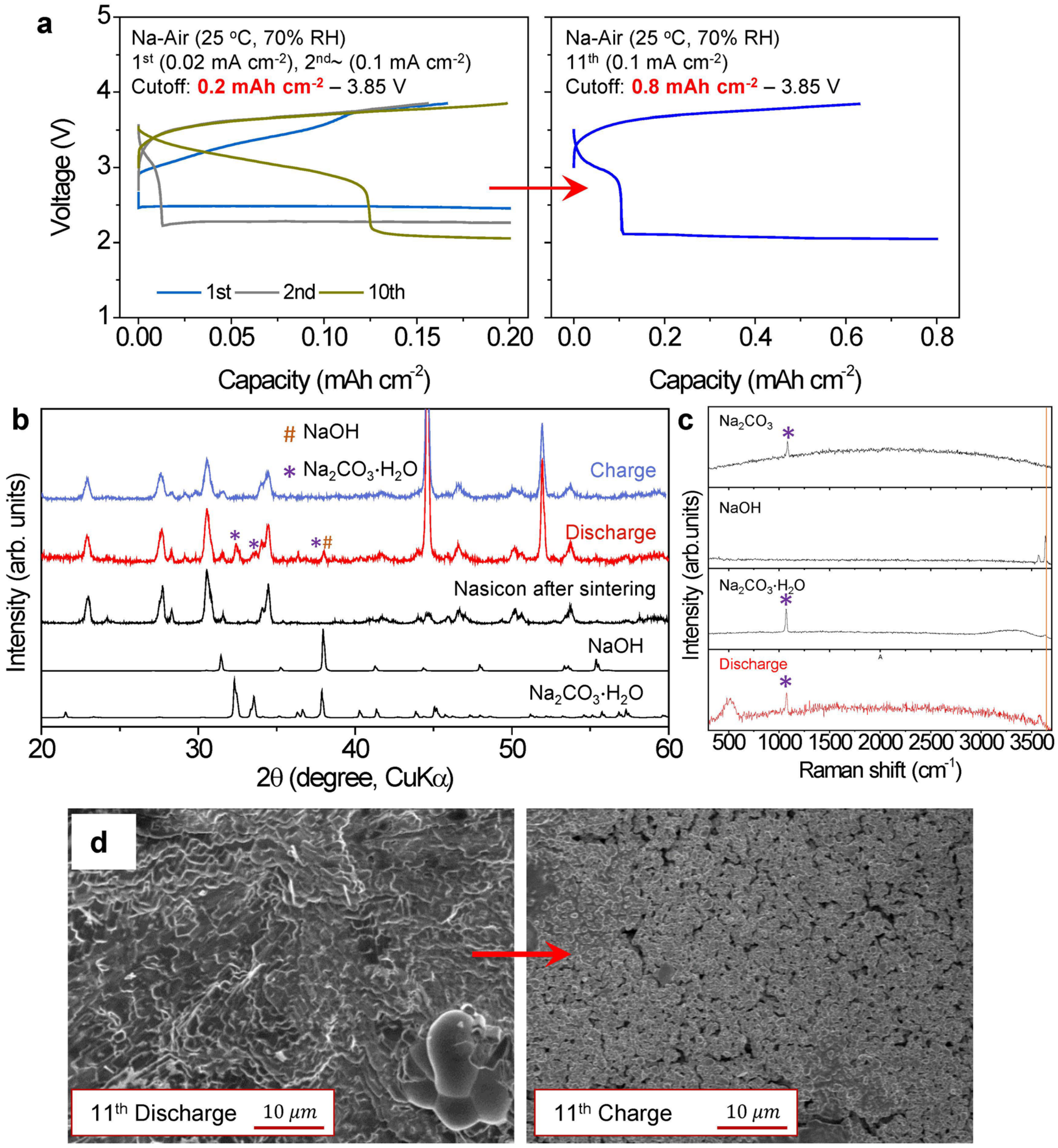

Figure S13. Ex-situ analysis of the air-electrode in 11th cycle of the SE-based Na-air cells. a-d, (a) Voltage profiles of the cell for ex-situ analyses at 0.02 and 0.1 mA cm^{-2} during 1st and subsequent cycles, respectively. (b) Ex-situ XRD spectra of the air-electrode in the 11th cycle. (c) Raman spectra of sodium compounds present in the air-electrode of the Na-air cell after 11th discharge. (d) SEM images (top view) of the air-electrode in the 11th cycle.

8-5. Evidence for supporting the presence of the absorbed water in the air-electrode

The presence of the absorbed H_2O can be confirmed by charging the cell to > 3.9 V. The cells were tested at 0.01 mA cm^{-2}. Only cell that was operated in air with 70% RH showed the long voltage plateau at 3.9 V. However, the cell that was operated in air with 40% RH did not exhibit the long voltage plateau at 3.9 V (Fig. S14a). At reduced discharge capacity (0.01 mAh cm^{-2}), the 3.9 V plateau did not appear (Fig. S14b). These results can imply that the increase in the absorbed water requires both NaOH, the discharge product, and a certain quantity of H_2O in air.

Also, in order to visualize the absorbed H_2O, SEM image of the air-electrode after vacuum drying was observed. As shown in Fig. S13, the cell was activated for 10 cycles, and then the cell was discharged to 0.8 mAh cm^{-2}. After that, the cell was disassembled and vacuum dried at 80 °C. Compared to the microstructure of the discharged air-electrode (Fig. S13d), rod-like morphology was observed instead of the blurred particle boundaries (Fig. S14c). The result clearly illustrates that the absorbed H_2O dissolves the discharge products leading to the formation of the blurred particle boundaries.

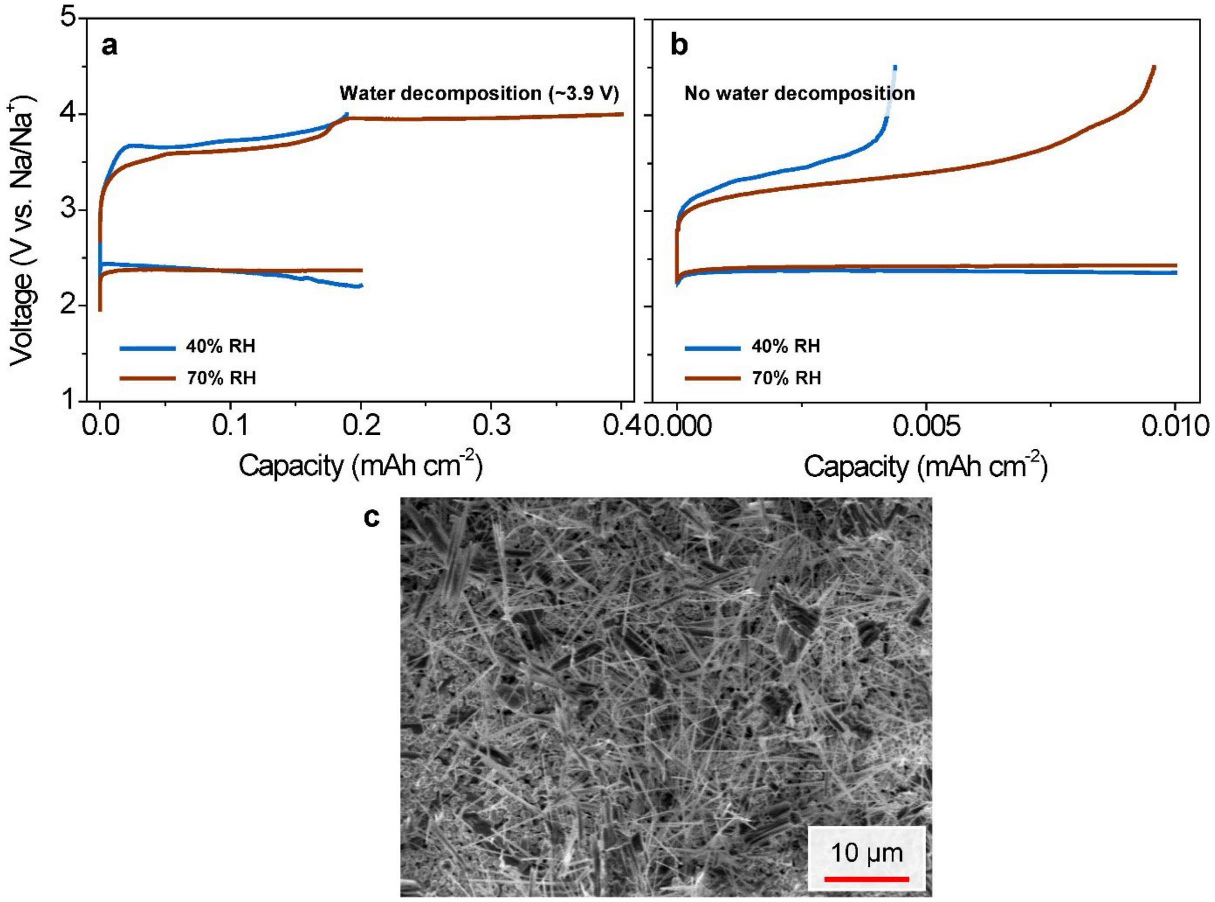

Figure S14. Voltage profiles of the SE-based Na-air batteries with varied discharge capacities and charge cut-off voltage under open air of 40% and 70% RH. a-b, Voltage profiles at 0.02 mA cm^{-2} within (a) 0.2 mAh cm^{-2} discharge - 4.0 V charge and (b) 0.01 mAh cm^{-2} discharge – 4.5 V charge. **c,** SEM images (top view) of the air-electrode vacuum-dried after the 11th discharge.

8-6. Negligible side reactions related to Ni such as $Ni(OH)_2$/NiOOH at ~ 3.4 V

The SE-based Na-air cell utilizes Ni current collector, and it is known that Ni can be transformed into $Ni(OH)_2$ in alkaline solution[7] and $Ni(OH)_2$/NiOOH redox reaction can be exhibited at ~3.2 V (vs. Na/Na^+).[8] Therefore, the possibility that Ni related reaction was included in the ~ 3.4 V plateau during discharge was confirmed (Fig. S15). When the Ni current

collector was investigated by XPS, Ni oxides as well as Ni metal was observed, which implies that a thin Ni oxides layer covers Ni particles (Fig. S15a). In addition, the effect of Ni content in the air-electrode was confirmed (Fig. S15b-d). The Ni infiltration process is performed for 2 times as explained experimetal section in the manuscript, and another cell by infiltrating Ni sources for 4 times was prepared. Even though the Ni content in the air-electrode become double, the ~3.4 V plateau doesn't increase as the Ni content. Combining these results, it can be concluded that the side reactions related with Ni might not be affect the appearance of the ~3.4 V plateau by following reason; the Ni particles barely transforms into $Ni(OH)_2$ due to the presence of Ni oxides layer as the previous study reported.[7]

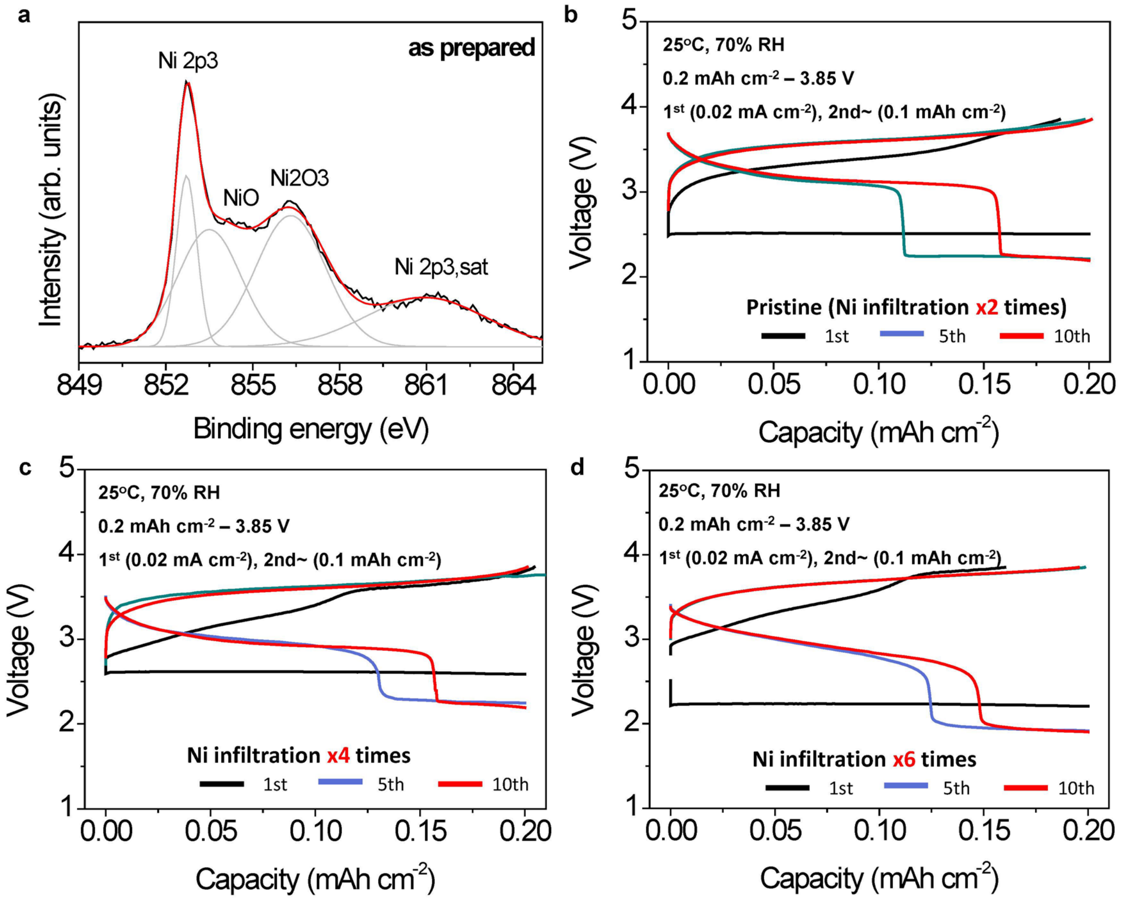

Figure S15. Exploration of electrochemical activity of Ni compounds. a, XPS analysis of the Ni current collector in the pristine SE-based Na-air cell revealed both Ni oxides and Ni metal. **b-d,** Comparison of voltage profiles of the SE-based Na-air cell: Ni infiltration process

performed for (b) twice (pristine cell), (c) four times and (d) six times, respectively. The cell tests were performed at 0.02 (1st cycle) and 0.1 mA cm^{-2} (subsequent cycles) within 0.2 mAh cm^{-2} (discharge) – 3.85 V (charge).

8-7. Cell tests based on the hybrid electrolytes (Aqueous electrolyte and solid electrolyte)

To further investigate how H_2O contributes to the activation of the electrochemically-reversible carbonate reactions (equations 5-6 in the manuscript), cell tests with the 'hybrid' electrolyte systems were performed by covering the stainless-steel plate and directly/intentionally filling the empty space of the air-electrode side with the hybrid electrolytes (1 mL) such as 0.6 M NaOH in DI water and 0.3 M Na_2CO_3 in DI water (Fig. S16a). The two hybrid electrolytes have the same amount Na ions in the aqueous solution. The cells firstly started with a charge process to decompose the Na compounds because they already have Na compounds dissolved in H_2O, as in the discharged state of the Nasicon SE-based Na-air battery (Fig. S16b, c). These cells were charged and then discharged to 0.2 mAh cm^{-2} at 0.2 mA cm^{-2}. Because the goal was to identify of types of discharge reactions by analyzing only the evolved gases from the decomposition of Na salts, three cells did not use any bubbling gases through the aqueous solutions, which are typically performed in common hybrid electrolyte system.[9-11] It should be noted that the cells with the hybrid electrolytes were close to the air.

The cell with the NaOH hybrid electrolyte (Fig. S16b) shows a main plateau at ~ 2.0 V during the 1st discharge, and the plateau at ~ 3.4 V increased only slightly during repeating cycling. The main plateau at ~ 2.0 V could be associated with a two-electron oxygen redox reaction at the cathode ($O_2 + H_2O + 2e^- \rightarrow HO_2^- + OH^-$, $E°$ = -0.08 V vs. SHE (standard

hydrogen electrode)) as has been reported in a hybrid Na-air cell with a similar cell configuration to the one used in this study.[11] The short plateau at ~ 3.4 V might be a result of formation of Na_2CO_3 by the exposure of NaOH to air during preparation and operation of the cell even with the closed condition. There is an additional small plateau at ~ 2.7 V during discharge. This potential may be a result of a cathodic reaction of hydrogen evolution ($2H^+ + 2e^- \rightarrow H_2$ (g), $E°$ = 0.00 V vs. SHE) that can occur in a hybrid Na-CO_2 system[9], but the possibility must be investigated further. In contrast, the cell with the Na_2CO_3 hybrid electrolyte (Fig. S16c) shows the discharge plateau at ~3.4 V and this plateau is extended during repeated cycling. The growth of the 3.4 V discharge plateau (~0.15 mAh cm^{-2} at the 10th cycle) in the cell with the Na_2CO_3 hybrid electrolyte is similar to the electrochemical behavior of the SE-based Na-air cell at the beginning of cycles.

In addition, we operated the cell in a CO_2-free air to understand the role of the CO_2 by preparing a Na – O_2 bubbled H_2O cell (Fig. S16d). The Na-air cell was operated under O_2 bubbled H_2O for 1 hr without any CO_2. The cells were discharged to 0.2 mAh cm^{-2} and charged up to 3.85 V at current densities of 0.02 for 1st cycle or 0.1 mA cm^{-2} for subsequent cycles. It did not exhibit the ~3.4 V reaction plateau, which is related to the reaction of $Na_2CO_3 \cdot xH_2O$, and the electrochemical activity was quickly degraded during cycles.

In consequence, it was observed that the new discharge plateau at ~ 3.4 V can be activated only if CO_2 gas is evolved and stored in H_2O during the charge, resulting in the increase in the CO_2 concentration. The CO_2 gas is about two orders of magnitude more soluble in H_2O than the O_2 gas is[12], so the CO_2 concentration nearby reaction sites inside of the air-electrode can be expected to increase drastically in the presence of absorbed H_2O. The similarity of the voltage curve and evolution with cycles in the SE-based Na-air cell to the cell with Na_2CO_3 hybrid electrolyte provides strong evidence that the electrochemical reaction of Na_2CO_3 in H_2O is

probably the dominant redox mechanism in the the SE-based Na-air battery, but further investigation to identify the electrochemical reactions occur at ~ 3.4 V during the discharge would be informative.

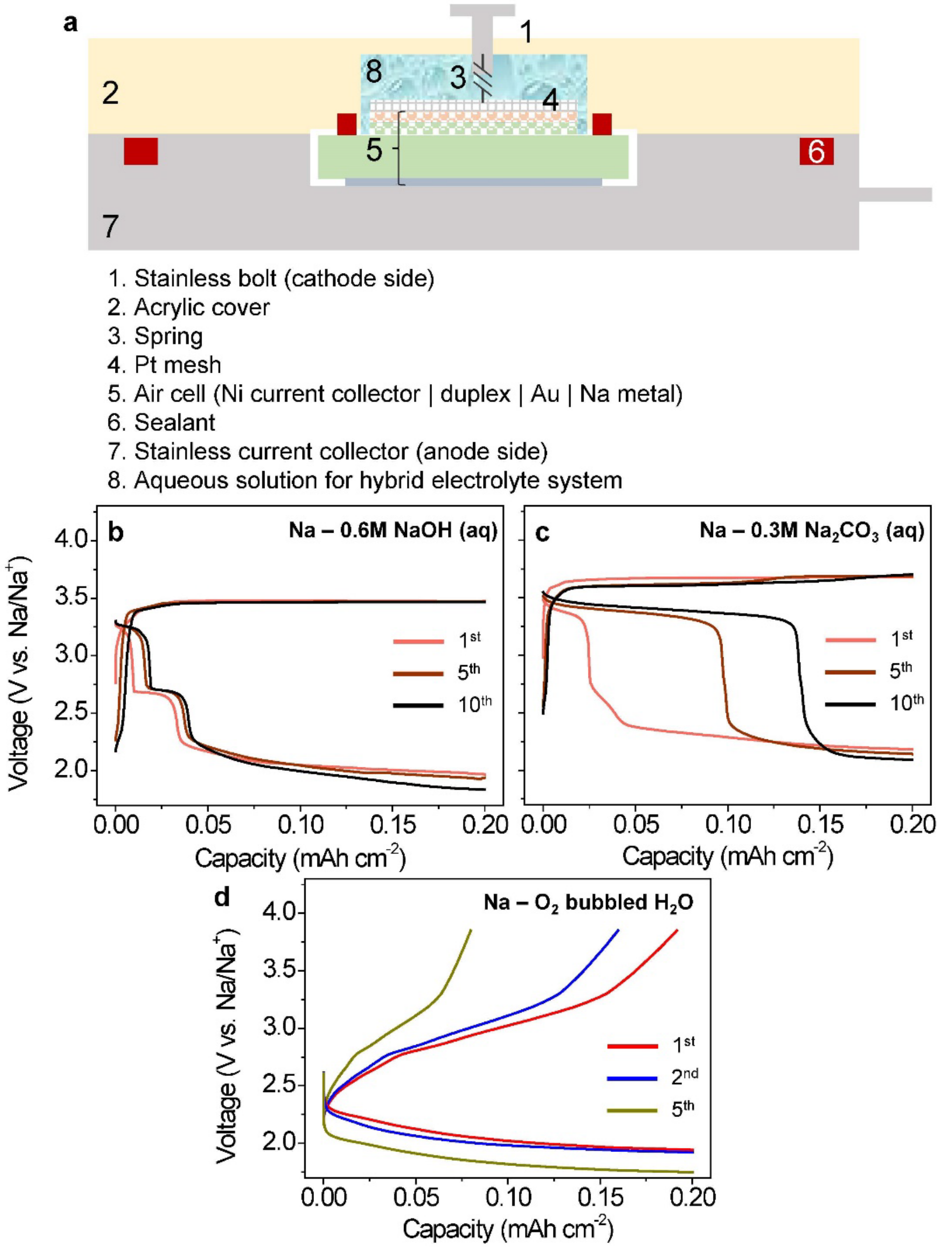

Figure S16. Clarifying the impact of H_2O and discharge products on voltage profile. **a,** Schematic diagram of the cell structure for the hybrid (aqueous + Nasicon) electrolyte Na-air cell. **b-c,** Voltage profiles of the cells with different hybrid electrolytes. (b) Na – 0.6M

NaOH (aq) and (c) Na – 0.3M Na_2CO_3 (aq) cells (Cut-off conditions of charge and discharge: 0.2 mAh cm^{-2} at 0.2 mA cm^{-2}) obtained at RT. **d,** Na – O_2 bubbled H_2O obtained at RT. (discharged to 0.2 mAh cm^{-2} and charged up to 3.85 V at 0.2 mA cm^{-2}).

8-8. Ex-situ Raman analysis for the hybrid Na-air based on Na_2CO_3 aqueous solution

In order to observe whether $Na_2CO_3{\cdot}xH_2O$ is electrochemically formed at ~ 3.4 V during discharge, the hybrid (aqueous + Nasicon solid electrolytes) Na-air cells with 0.3M Na_2CO_3 aqueous solution were tested (Fig. S17). It should be noted that the as-prepared hybrid Na-air cell is very similar with the discharged state of the SE-based Na-air cell. After the cell test, the hybrid electrodes with the aqueous solutions were collected and then dried with vacuum. Then, the dried powder electrodes were characterized by Raman spectra. When the hybrid cell was discharged to 2.5 V, the Raman band was observed at only ~1080 cm^{-1}, which is related to the Na_2CO_3, whereas the additional band at ~ 3633 cm^{-1} corresponding anhydrous NaOH was observed in the cell discharged to 1.0 mAh cm^{-2}.[6] These results supports that the dissolved CO_2 into the absorbed water can induce the formation of Na_2CO_3 and the NaOH in subsequent discharge and the Na_2CO_3 formation reaction has higher voltage than the NaOH formation reaction. As a result, the formation of the Na_2CO_3 in the Na-air cell can increase the discharge reaction voltage of Na-air battery after 2nd cycles.

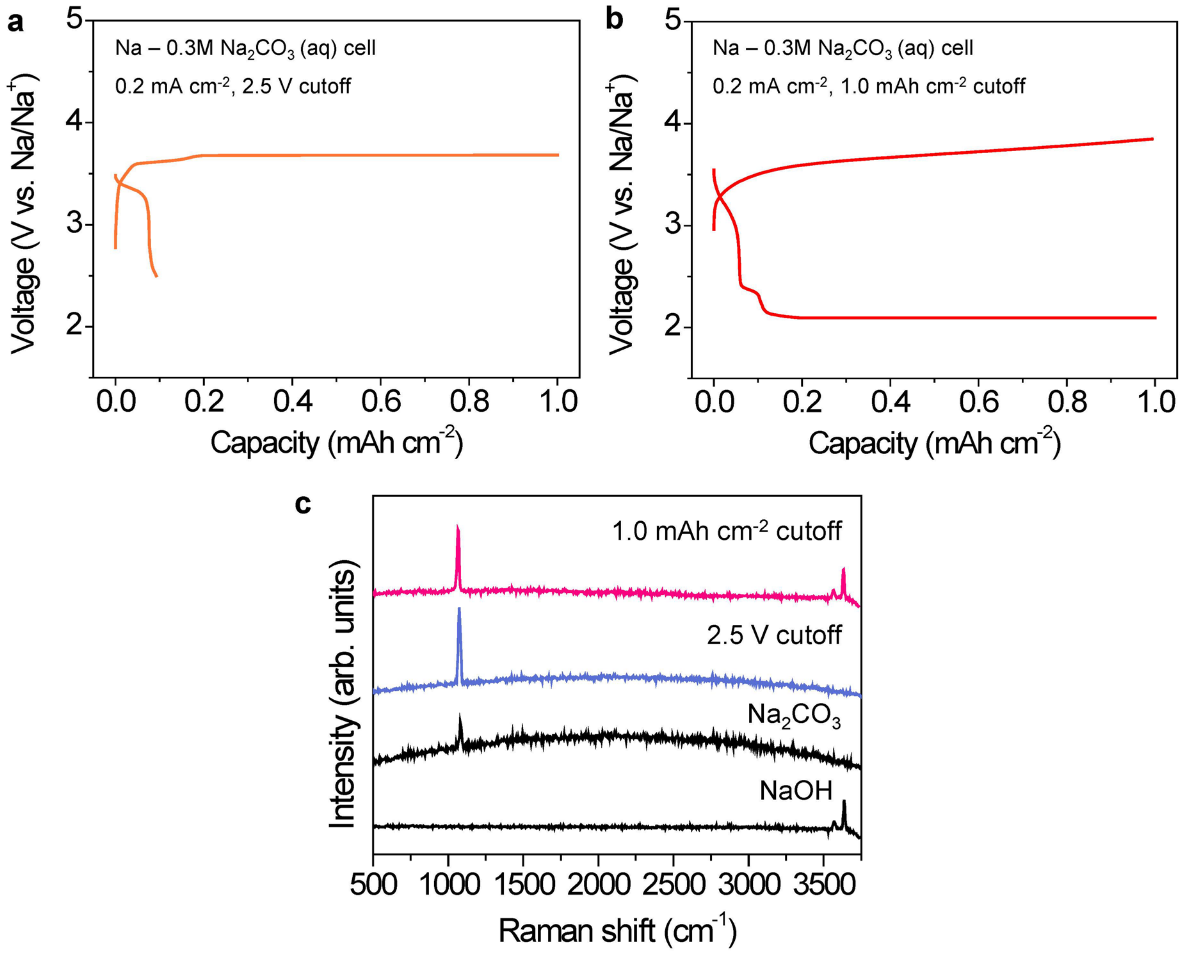

Figure S17. Verifying discharge products of the hybrid electrolyte (Na_2CO_3 aqueous solution + Nasicon) cells. a-b, Voltage profiles of the hybrid electrolyte cells discharged to (a) 2.5 V and (b) 1.0 mAh cm^{-2}, respectively. After charging to 1.0 mAh cm^{-2}, the cells were discharged at a current density of 0.2 mA cm^{-2}. **c,** Ex-situ Raman spectra of the vacuum-dried Na_2CO_3 solutions from the cells. It should be noted that the charged state of the Na-air cell closely resembles that of the hybrid Na-air cell with Na_2CO_3 aqueous solution.

8-9. Necessity of the NaOH reaction at the end of each discharge for reversible carbonates reaction

At the end of each discharge, the small portion of the NaOH reaction always exhibited at ~ below 2.0 V after 2nd cycle (Fig. 2-4 in the manuscript). It should be noted that this reaction at below 2 V after 2nd cycle is the same reaction at ~ 2.5 V at 1st discharge considering the higher current density in 2nd cycle than 1st cycle. To understand the role of this NaOH reaction on the electrochemical reaction of $Na_2CO_3 \cdot xH_2O$, an additional cycle test was performed with different discharge cut-off voltage to activate only the reactions of the carbonates. At first 5 cycles, the cell was discharged to 0.2 mAh cm^{-2} and charged to 3.85 V to sufficiently activate the carbonate reactions at ~ 3.4 V (Fig. S18a). After that, the cell was intentionally discharged to 2.5 V not to activate the NaOH reaction at ~ below 2.0 V, and then the capacity of the cell was gradually decreased by repeating cycles (Fig. S18b). Also, when the cell was discharged to 0.2 mAh cm^{-2} again, which can allow the NaOH reaction below 2.0V, the discharge capacity at ~ 3.4 V was recovered (Fig. S18c). The result suggests that the activation of the NaOH reaction at the end of the discharge in each cycle is essential to maintain reversibly the electrochemical carbonates reactions in subsequent cycles.

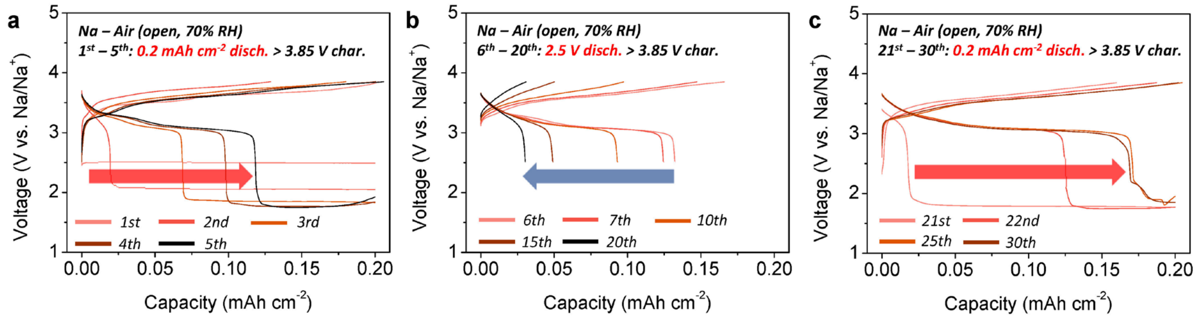

Figure S18. Voltage profiles under different discharge cut-off conditions in the SE-based Na-air cell. a-c, Voltage profiles of the cell (a) discharged to 0.2 mAh cm^{-2} during 1st to 5th cycles, (b) a cut-off voltage of 2.5 V applied from 6th to 20th cycles (without NaOH reaction in the discharge), and (c) discharged to 0.2 mAh cm^{-2} again from 21st to 30th cycles.

The cell was operated in open air with 70% RH at 25 °C, with initial cycle current density at 0.02 mA cm^{-2}, followed by 0.1 mA cm^{-2} in subsequent cycles.

8-10. Calculation of maximum discharge capacity based on pore volume in the air-electrode

To understand how the in-situ formed catholyte, which is formed by dissolving the discharge product NaOH in 1st discharge into the absorbed water, affects the discharge process of the SE-based Na-air cell, the cell was fully discharged (Fig. S19). The air-electrode consists of the two porous layers: porous Nasicon layer with nano-sized Ni particles and Ni current collector (Figure 1 in the manuscript). However, the thickness of the Ni current collector alone could not be measured easily, so an Au layer was used as the current collector instead of Ni for the full discharge test. The Au layer was laminated by printing Au paste (Tanaka) and drying at 700 °C for 10 min in ambient air, and the thickness of the Au layer (6 μm) was measured by EDS (Energy Dispersive spectrometry) mapping (Fig. S19a, b). Dimensions of the Nasicon and Ni composite layer were ~ 44 μm (thickness) and ~ 0.5 cm^2 (area) so that total volume of the layer was 2.2 x 10^{-3} cm^3. Considering that negligible amounts of Ni were included in this layer, and the weight of the Nasicon in this layer was 2.2 mg with a density of 3.2 g cm^{-3}, the pore volume of the layer was calculated as 1.5 x 10^{-3} cm^3 from the difference between measured and expected weight. Also, considering the Au layers' dimensions (4 μm x 0.5 cm^2), weight (2.6 mg), and density (19.3 g cm^{-3}), the calculated pore volume of the Au layer was 6.5 x 10^{-5} cm^3. Maximum capacity by filling the pore with NaOH (density = 2.02 g cm^{-3}), Na_2CO_3 (density = 2.33 g cm^{-3}), and $Na_2CO_3 \cdot H_2O$ (density = 2.26 g cm^{-3}) were calculated (Table S3). When the cell was fully discharged, it achieved ~ 6.3 mAh cm^{-2} discharge capacity, which is around 1.5 times higher than calculated the maximum capacity (Fig. S19c and Table S3), and Na compounds were also found in the Pt mesh (Fig. S20), that was not in contact with any ionic

conductor (4 in Fig. S4) in the pristine. These results indicate that the in-situ formed catholyte can act as a new ionic conductor that can increase the area of the TPB and improve the discharge capacity of the cell.

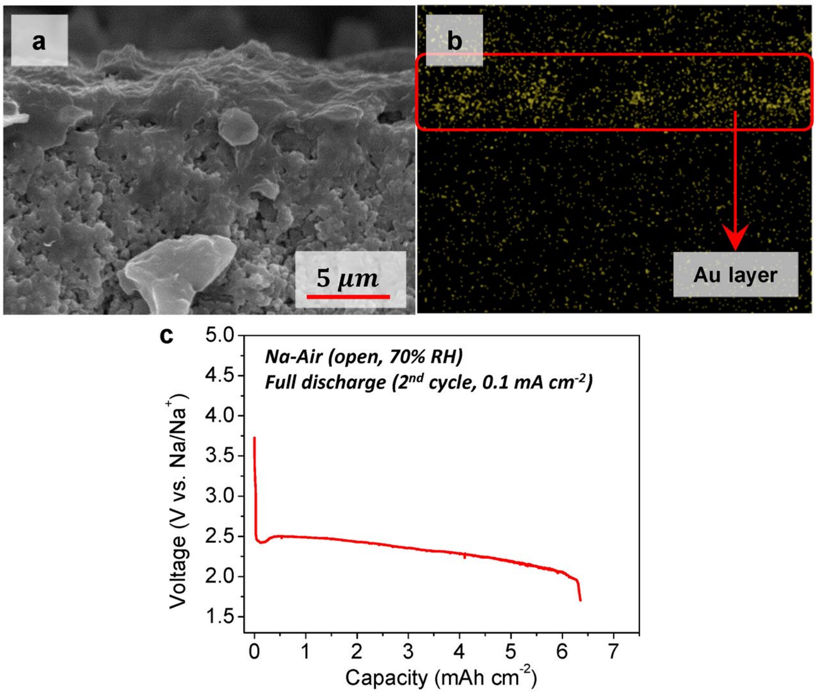

Figure S19. Full discharge capacity evaluation of the SE-based Na-air cells. a-b, SEM and EDS analyses of the SE-based Na-air cell for the full discharge test. The thickness of the Au current collector was approximated using EDS measurements. **c,** Voltage profile for the full discharge capacity test with the cell discharged to 1.5 V at a current density of 0.1 mA cm^{-2}. We note that the cell was pre-activated for one cycle, discharging to 0.2 mAh cm^{-2} and charging up to 3.85 V, at 0.02 mA cm^{-2}.

Table S3. Maximum capacity to completely fill the pore in the air electrodes

Maximum capacity (mAh cm^{-2})	Nasicon & Ni layer	Au layer

NaOH	4.04	0.18
Na_2CO_3	3.52	0.15
$Na_2CO_3{\cdot}H_2O$	2.92	0.13

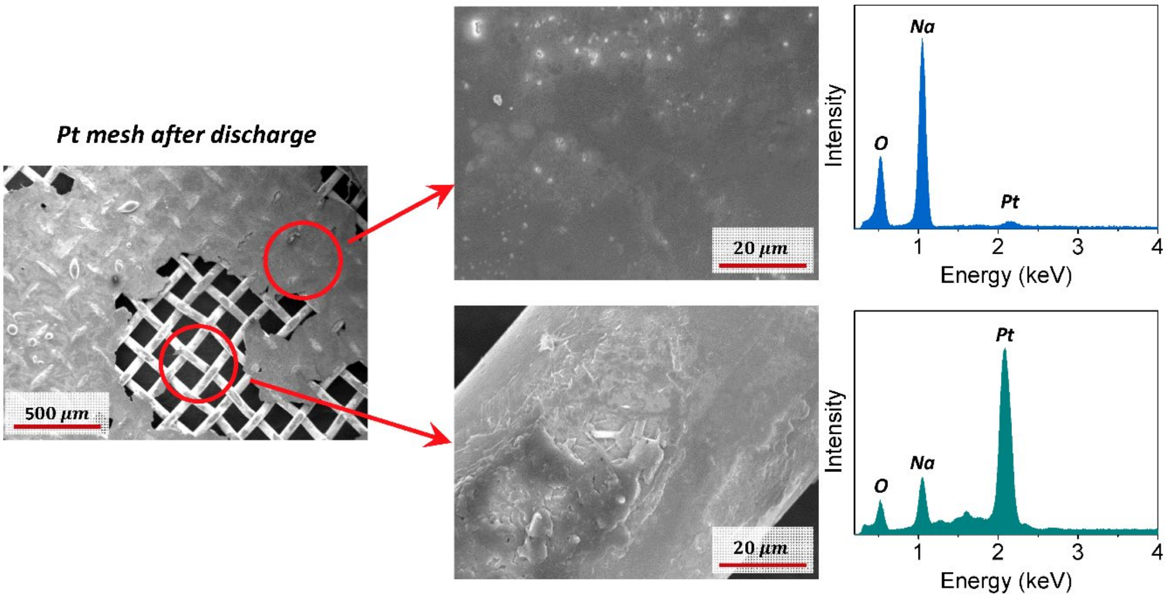

Figure S20. SEM and EDS images of Pt mesh that does not have any contact with an ionic conductor after the full discharge test (Fig. S19).

9. Results of cycle and rate capability tests under different RH conditions

9-1. A procedure of the rate capability test in air with 70% RH

The cells were activated by cycling 10 times in air with 70% RH before the rate capability test in air with 70% RH as well as 40% RH (Fig. S21a). Current densities in this process were 0.02 mA cm^{-2} during the 1st cycle and 0.1 mA cm^{-2} during subsequent cycles. After that, the rate capability test was conducted by cycling five times at each current density after the activation process (Fig. S21b).

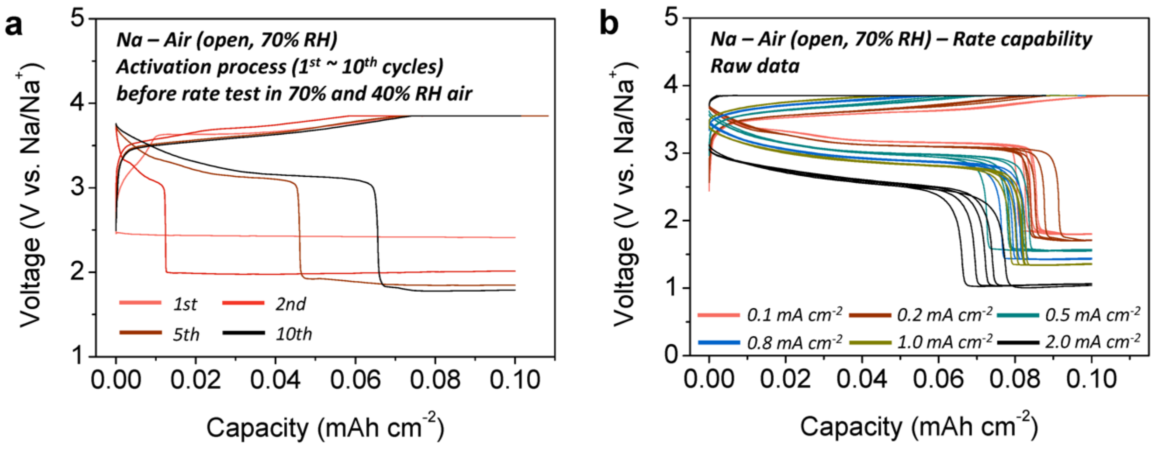

Figure S21. Rate capability test of the SE-based Na-air cells in ambient air with 70% RH. a, 1st ~ 10th cycles as the activation process performed in air with 70% RH before rate capability tests in air with 70% RH (Fig. 4c, f in manuscript). The current densities at 1st and the other cycles were 0.02 and 0.1 mA cm^{-2}, respectively. **b,** Raw data of the rate capability test conducted in air with 70% RH (Fig. 4c in manuscript). All tests in Fig. S21 were conducted at 25 °C. The cells were discharged to 0.1 mAh cm^{-2} and charged in CCCV mode; after charging the cell to 3.85 V at constant current, the voltage was held at 3.85 V until the current reached < 30 % of the applied current.

9-2. Necessity of the activation process to operate the cells in air with low RH (= 40%)

When the voltage curve was measured in air with 40% RH only, the plateau at ~ 3.4 V, which is related to the electrochemical sodium carbonates reaction, grew slightly even after repeating cycles (Fig. S22a) compared to that in air with 70% RH. This indicates that the $Na_2CO_3 \cdot xH_2O$ reactions in air with 40% RH are activated very slowly. Therefore, the cycle and rate capability tests in air with 40% RH were proceeded after the activation process, which is cycled for 10 cycles in air with 70% RH as shown in Fig. S21a. Raw data of the rate capability test in air with 40% RH are also included in Fig. S22b.

The SE-based Na-air cell after the pre-activating process also operated in ambient air where relative humidity is lower than 40% (Fig. S23). As shown in the figure, the variance of coulombic efficiency was similar to that of the relative humidity. It implies that the amount of H_2O in the air-electrode changed by the humidity strongly affects the kinetics. At 40th cycle, the relative humidity decreased from 70% to ~35%. The 2nd discharge plateau was decreased by the ~3.4 V plateau was maintained. However, when the relative humidity decreased to 21% (65th cycle), the capacity of the ~3.4 V plateau during discharge was reduced and the two plateaus were observed clearly during charge (Fig. S23b). It can support that lowering the absorbed H_2O under low humidity can affect the chemical reaction between NaOH (discharge product) and CO_2 and the kinetics.

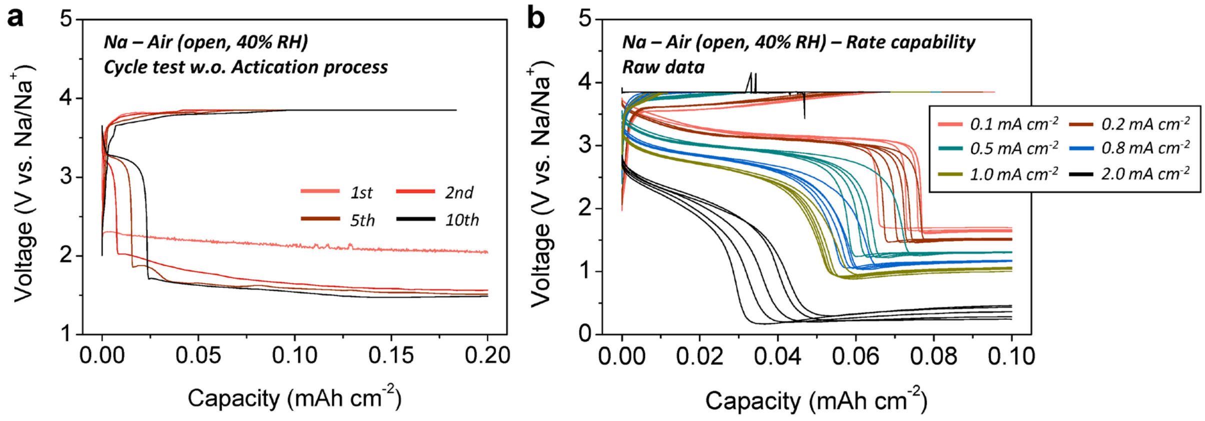

Figure S22. Cyclability and rate capability tests of the SE-based Na-air cells in ambient air with 40% RH. a, Cycle test conducted in air with only 40% RH without the activation process. The current densities for the 1st and the subsequent cycles were 0.02 and 0.1 mA cm^{-2}, respectively. **b,** Raw data of rate capability tests in air with 40% RH following the activation process (Figure 4f in manuscript). All tests depicted in Fig. S22 were conducted at 25 °C. The cells were discharged to 0.1 mAh cm^{-2} and charged in CCCV mode; after

charging the cell to 3.85 V at constant current, the voltage was held at 3.85 V until the current reached < 30 % of the applied current.

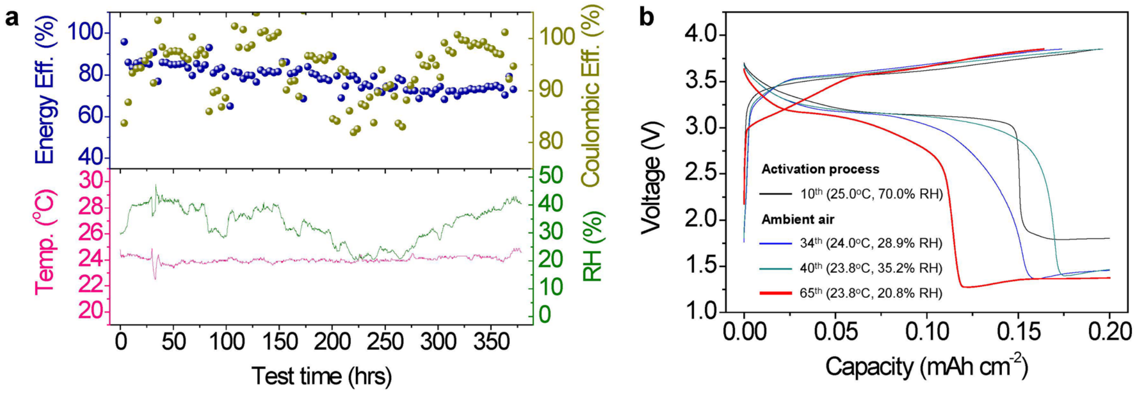

Figure S23. The electrochemical performance of the SE-based Na-air cell operated in 'ambient air' after pre-cycling at 70% RH at 25°C. a, Variations in the cell performance and ambient air conditions during cycle test. **b,** Voltage profiles of the SE-based Na-'ambient air' cell in different relative humidity conditions. After pre-activating the cell in air with 70% RH at 25 °C (Fig. 2a in the manuscript), the cell was discharged to 0.2 mAh cm^{-2}, and charged to 3.85 V at a current density of 0.1 mA cm^{-2}.

10. Degradation of the Na metal anode

Sudden voltage drop during the discharge in cycles was observed when the Nasicon SE-based Na-air cell was failed as shown in Fig. S24. The cell test was conducted in air with 70% RH at 25 °C. The cell was discharged to 0.2 mAh cm^{-2}, then charged to 3.85 V. The current density was 0.1 mA cm^{-2}. After the death of the cell, the Na metal anode was discolored indicating the contamination of the Na metal. As a result, we inferred that the contamination of Na metal severely affected the cycle life of the cell (Fig. S24a). When fresh Na metal was replaced with the discolored Na metal in the dead cell, its electrochemical characteristics were

recovered (Fig. S24b). This indicates that the contamination of Na metal can be a main reason of the cell failure.

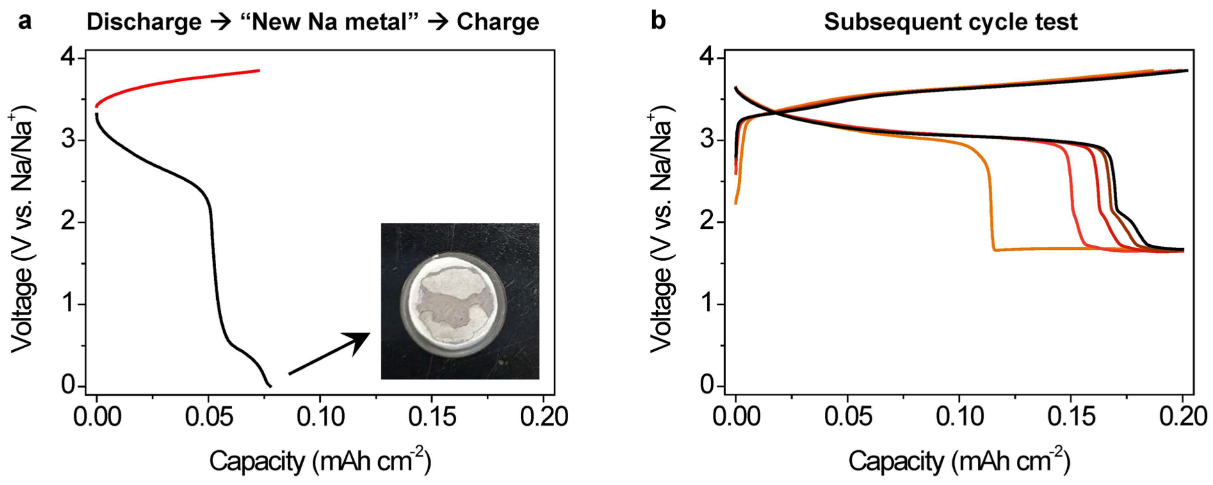

Figure S24. Impact of Na metal contamination on the degradation of the SE-based Na-air batteries. a, Voltage profile showing abrupt cell failure during discharge (inset: anode side of the cell after cell failure). **b,** Voltage profiles after replacing the discolored Na metal with fresh Na metal. The cell test was conducted in air with 70% RH at 25 °C. The cell was discharged to 0.2 mAh cm^{-2}, and then charged to 3.85 V at a current density of 0.1 mA cm^{-2}.

11. Theoretical energy density of the Li/Na-air batteries based on discharge products

Energy densities of the electrochemical reactions in Li/Na-air (O_2) batteries were calculated from the thermodynamic potential and theoretical capacity of discharge products (Table S4). The discharge products in the SE-based Na-air cell have high theoretical potential, and therefore the SE-based Na-air cell shows higher energy densities than the cell with Li/Na superoxides (MO_2).

Table S4. Energy densities and theoretical potential of Li/Na-air batteries based on various discharge products

Type	Reaction	Energy density (Wh $kg^{-1}_{disch.\ product}$)	Potential (V)
**Li-O_2**	$Li + O_2 \rightarrow LiO_2$	1693.16	2.61
	$2Li + O_2 \rightarrow Li_2O_2$	3458.18	2.96
	$2Li + 0.5O_2 \rightarrow Li_2O$	5220.11	2.91
**Na-O_2**	$Na + O_2 \rightarrow NaO_2$	1106.40	2.27
	$2Na + O_2 \rightarrow Na_2O_2$	1601.66	2.33
	$2Na + 0.5O_2 \rightarrow Na_2O$	1686.47	1.95
**Na-Air**	$Na + 0.5H_2O$ (g) $+ 0.25O_2 \rightarrow NaOH$ (s)	1842.73	2.75
	$Na + CO_2 + 0.5O_2 \rightarrow Na_2CO_3$ (s)	1704.35	3.37
	$Na + CO_2 + 0.5O_2 + H_2O$ (g) $\rightarrow Na_2CO_3 \cdot H_2O$ (s)	1482.68	3.43

12. Comparison of the electrochemical performances between the SE-based Na-air battery and the Li/Na-air(O_2) battery reported in previous studies

The difference in potential between discharge and charge, and working current density of the SE-based Na-air battery were compared to the other systems (Table S5). The operating temperature was 25 °C or room temperature. The SE-based Na-air battery was tested under a reasonable level of working conditions.

Table S5. Comparison of the electrochemical characteristics of the Li/Na–air(O_2) batteries in this study and previous reports

Type	Reaction	Potential gap (V)	Current density (mA cm^{-2})	Current density (mA g^{-1})	Ref.
**Li – air (O_2)**	$Li + O_2 \rightarrow LiO_2$	1.25		100	13
	$2Li + O_2 \rightarrow Li_2O_2$	2.0	0.02		14
		1.5		500	4
		1.5		2000	15
		1.1	0.3		16
		> 1.5	0.1		17
	$2Li + 0.5O_2 \rightarrow Li_2O$ (150 °C)	0.2	0.1		17
**Na – air (O_2)**	$Na + O_2 \rightarrow NaO_2$	0.1	0.12		14
		0.1	0.2		18
		0.1	0.2	67	19
		0.15	0.05		20
	$2Na + O_2 \rightarrow Na_2O_2$	1.25		300	21
	$Na + 0.5H_2O$ (g) $+ 0.25O_2 \rightarrow NaOH$ (s) **(ambient air)**	1.0	0.1		This work
		-		100	22
	$Na + CO_2$ (g) $+ 0.5O_2 \rightarrow Na_2CO_3$ (s) **(ambient air)**	0.5	0.1		This work
		-		100	22
	$Na + CO_2$ (g) $+ 0.5O_2 + H_2O$ (g) $\rightarrow Na_2CO_3{\cdot}H_2O$ (s) **(ambient air)**	0.5	0.1		This work

Reference

1 Tsai, C.-L. *et al.* $Li_7La_3Zr_2O_{12}$ Interface Modification for Li Dendrite Prevention. *ACS Appl. Mater. Interfaces* **8**, 10617-10626 (2016).

2 Rafael, S.-P. *et al.* Sodium–gold binaries: novel structures for ionic compounds from an ab initio structural search. *New J. Phys.* **15**, 115007 (2013).

3 Tang, S. *et al.* Stable Na Plating and Stripping Electrochemistry Promoted by In Situ Construction of an Alloy-Based Sodiophilic Interphase. *Adv. Mater.* **31**, 1807495 (2019).

4 Asadi, M. *et al.* A lithium–oxygen battery with a long cycle life in an air-like atmosphere. *Nature* **555**, 502 (2018).

5 Walrafen, G. E. & Douglas, R. T. W. Raman spectra from very concentrated aqueous NaOH and from wet and dry, solid, and anhydrous molten, LiOH, NaOH, and KOH. *J. Chem. Phys.* **124**, 114504 (2006).
6 Yin, S., Zeng, Y., Li, C., Chen, X. & Ye, Z. Investigation of Sm0.2Ce0.8O1.9/Na2CO3 Nanocomposite Electrolytes: Preparation, Interfacial Microstructures, and Ionic Conductivities. *ACS Appl. Mater. Interfaces* **5**, 12876-12886 (2013).
7 Trafela, Š., Zavašnik, J., Šturm, S. & Rožman, K. Ž. Formation of a Ni(OH)2/NiOOH active redox couple on nickel nanowires for formaldehyde detection in alkaline media. *Electrochim. Acta* **309**, 346-353, (2019).
8 Park, S., Khan, Z., Shin, T. J., Kim, Y. & Ko, H. Rechargeable Na/Ni batteries based on the Ni(OH)2/NiOOH redox couple with high energy density and good cycling performance. *J. Mater. Chem. A* **7**, 1564-1573, (2019).
9 Kim, C. *et al.* Efficient CO_2 Utilization via a Hybrid Na-CO_2 System Based on CO_2 Dissolution. *iScience* **9**, 278-285 (2018).
10 Hwang, S. M., Go, W., Yu, H. & Kim, Y. Hybrid Na–air flow batteries using an acidic catholyte: effect of the catholyte pH on the cell performance. *J. Mater. Chem. A* **5**, 11592-11600 (2017).
11 Hayashi, K., Shima, K. & Sugiyama, F. A Mixed Aqueous/Aprotic Sodium/Air Cell Using a NASICON Ceramic Separator. *J. Electrochem. Soc.* **160**, A1467-A1472 (2013).
12 Kolev, N. I. in *Multiphase Flow Dynamics 4: Turbulence, Gas Adsorption and Release, Diesel Fuel Properties* 209-239 (Springer Berlin Heidelberg, 2012).
13 Lu, J. *et al.* A lithium–oxygen battery based on lithium superoxide. *Nature* **529**, 377 (2016).
14 Hartmann, P. *et al.* A rechargeable room-temperature sodium superoxide (NaO_2) battery. *Nat. Mater.* **12**, 228-232 (2013).
15 Wang, L. *et al.* A Li–Air Battery with Ultralong Cycle Life in Ambient Air. *Adv. Mater.* **30**, 1704378 (2018).
16 Zhu, X. B., Zhao, T. S., Wei, Z. H., Tan, P. & An, L. A high-rate and long cycle life solid-state lithium–air battery. *Energy Environ. Sci.* **8**, 3745-3754 (2015).
17 Xia, C., Kwok, C. Y. & Nazar, L. F. A high-energy-density lithium-oxygen battery based on a reversible four-electron conversion to lithium oxide. *Science* **361**, 777-781 (2018).
18 Bender, C. L., Hartmann, P., Vračar, M., Adelhelm, P. & Janek, J. On the Thermodynamics, the Role of the Carbon Cathode, and the Cycle Life of the Sodium Superoxide (NaO_2) Battery. *Adv. Energy Mater.* **4**, 1301863 (2014).
19 Zhao, N., Li, C. & Guo, X. Long-life Na–O_2 batteries with high energy efficiency enabled by electrochemically splitting NaO_2 at a low overpotential. *Phys. Chem. Chem. Phys.* **16**, 15646-15652 (2014).
20 Xia, C., Black, R., Fernandes, R., Adams, B. & Nazar, L. F. The critical role of phase-transfer catalysis in aprotic sodium oxygen batteries. *Nat. Chem.* **7**, 496 (2015).
21 Liu, W., Sun, Q., Yang, Y., Xie, J.-Y. & Fu, Z.-W. An enhanced electrochemical performance of a sodium–air battery with graphene nanosheets as air electrode catalysts. *Chem. Commun.* **49**, 1951-1953 (2013).
22 Sun, Q. *et al.* Toward a Sodium–"Air" Battery: Revealing the Critical Role of Humidity. *J. Phys. Chem. C* **119**, 13433-13441 (2015).
